# Clinical decision rules, spinal pain classification and prediction of treatment outcome: A discussion of recent reports in the rehabilitation literature

**DOI:** 10.1186/2045-709X-20-19

**Published:** 2012-06-22

**Authors:** Jeffrey J Hebert, Julie M Fritz

**Affiliations:** 1 Faculty of Health Sciences, Murdoch University, Murdoch, WA, Australia; 2 Department of Physical Therapy, University of Utah and Intermountain Healthcare, Salt Lake City, UT, USA

**Keywords:** Diagnosis, Clinical prediction rule, Clinical decision making, Low back pain, Neck pain, Back pain

## Abstract

Clinical decision rules are an increasingly common presence in the biomedical literature and represent one strategy of enhancing clinical-decision making with the goal of improving the efficiency and effectiveness of healthcare delivery. In the context of rehabilitation research, clinical decision rules have been predominantly aimed at classifying patients by predicting their treatment response to specific therapies. Traditionally, recommendations for developing clinical decision rules propose a multistep process (derivation, validation, impact analysis) using defined methodology. Research efforts aimed at developing a “diagnosis-based clinical decision rule” have departed from this convention. Recent publications in this line of research have used the modified terminology “diagnosis-based clinical decision guide.” Modifications to terminology and methodology surrounding clinical decision rules can make it more difficult for clinicians to recognize the level of evidence associated with a decision rule and understand how this evidence should be implemented to inform patient care. We provide a brief overview of clinical decision rule development in the context of the rehabilitation literature and two specific papers recently published in Chiropractic and Manual Therapies.

## 

Healthcare has undergone an important paradigm shift toward evidence based practice; an approach thought to enhance clinical decision making by integrating the best available evidence with clinical expertise and the preferences of patients.[1] Ultimately, the goal of evidence based practice is to improve healthcare delivery. However, the translation of scientific evidence into practice has proven a challenging endeavour.

Clinical decision rules (CDRs), also known as clinical prediction rules, are an increasingly common presence in the rehabilitation literature. These are tools designed to inform clinical decision-making by identifying potential predictors of diagnostic test outcome, prognosis or therapeutic response.[2,3] In the rehabilitation literature, CDRs are most commonly used to predict a patient’s response to treatment and have been proposed as a means of identifying clinically relevant subgroups of patients presenting with otherwise heterogeneous disorders such as non-specific neck[4] or low back pain[5,6] and this is the perspective on which we intend to focus.

The ability to classify or subgroup patients with heterogeneous disorders such as spinal pain has been highlighted as a research priority[7,8] and consequently, the focus of much research effort. The appeal of such classification approaches is their potential for improved treatment efficiency and effectiveness by matching patients with optimal therapies. In the past, patient classification has relied on implicit approaches founded in tradition or unsystematic observations. The use of CDRs to inform classification is one attempt at a more evidence driven approach, less dependent on unfounded theory.

CDRs are developed in a multistep process involving studies of derivation, validation and analysis of impact with each having a defined purpose and methodological criteria.[2,9] As with all forms of evidence used to make decisions about patients, attention to appropriate study methodology is critical to assessing the potential benefits of implementation. While a comprehensive discussion of CDR methodology is beyond the scope of this commentary, we seek to present a brief overview of CDR development in the context of the rehabilitation literature and two specific papers recently published in Chiropractic and Manual Therapies.

## CDR derivation

The purpose of CDR derivation studies is to identify potentially important predictors of response to therapy and combine important predictors into a preliminary decision rule. Commonly, variables to be considered at this step are included from the history, physical examination and other forms of testing. Multivariate statistical analyses (*e.g.*, logistic regression) are then used to identify relationships between different clusters of variables and the outcome of interest (*e.g.*, decreased pain, increased function).

There has been much discussion and debate regarding the optimal research design of derivation studies.[10-13] Many derivation studies have been single-arm studies, meaning all patients receive the same therapy within the study. A single-arm study design for deriving a CDR has several advantages, foremost among these is that this design requires fewer resources and can generally be completed more rapidly than a two-arm study design such as a randomized trial. The shortcoming of a single-arm design, however, is the inability to distinguish true predictor variables (*i.e.*, effect modifiers) from those generally associated with a favourable prognosis unrelated to the treatment received.

For example, Flynn and colleagues[6] used a single arm design to identify those demographic, historical or physical examination factors associated with a favourable clinical outcome among patients with low back pain undergoing treatment consisting of spinal manipulative therapy and range of motion exercises. The study results identified five variables associated with a favourable treatment response:

1) Pain duration of less than 16 days

2) Fear avoidance beliefs work subscale score less than 19

3) At least one hip with greater than 35° internal rotation range of motion

4) Hypomobility of the lumbar spine

5) No symptoms distal to the knee

When at least 4 of these factors were present (*i.e.*, positive status on the rule), the reported positive likelihood ratio was approximately 24, meaning that individuals with low back pain meeting this criteria had a 95% likelihood of achieving clinical success with spinal manipulative therapy.

However, since an experimental study design was not used to validate these relationships, it was not possible from this derivation study to determine if these variables were predictors of treatment response specific to spinal manipulation (*i.e.*, treatment effect modifiers) or more generally indicative of a patient with a favourable prognosis regardless of management. Therefore, while in some cases it may be appropriate for clinicians to use data from derivation studies to inform clinical decision-making, CDRs must undergo the rigors of full validation (*e.g.*, testing in a randomized clinical trial) prior to widespread implementation.[2]

## CDR validation

The validation of a potential CDR is a critical step in its development and is requisite to broad implementation. There are several reasons for this. First, the associations between predictor variables and the outcome of interest identified at the derivation stage could have resulted from chance or may simply represent the identification of positive prognostic factors. Second, the predictor variables may be unique to the participants, clinicians or context of the derivation study and these relationships would therefore not be present in other circumstances. Third, the implementation of the CDR may fail due to problems related to the feasibility of its application.

The appropriate approach for examining the validity of a CDR employs an experimental study design to examine for statistical interaction (*i.e.*, treatment effect modification) between the potential predictor variables and the treatment received. To validate a CDR for predicting treatment response, cohorts of patients who do and do not meet the CDR criteria would be randomly assigned to receive the treatment specific to the CDR or an alternative approach, which could be no treatment. This design permits an assessment of whether the CDR identifies a group of patients who respond to a specific intervention, but not to an alternative treatment.

Returning to our example of the CDR for spinal manipulative therapy, Childs *et al.*[14] examined the validity of the rule using a two-arm randomized trial. The authors investigated the treatment outcomes experienced by patients with low back pain, classified based upon their status on the CDR who were randomly assigned to receive spinal manipulation plus exercise or exercise alone.[14] The results indicated that CDR positive patients experienced a better outcome with spinal manipulation and exercise (*i.e.*, when CDR status was matched to therapy) as compared to those receiving exercise only. Furthermore, the matched patients had better clinical outcomes than CDR negative patients undergoing an exercise program with or without spinal manipulation. These results provided evidence supporting the validity of the CDR criteria. However, as the sample population was similar to that examined in the derivation study, this result was considered evidence of narrow validation. Additional knowledge regarding the validity of this CDR would be gained through a more comprehensive investigation of its validity.

## CDR analysis of impact

Ultimately, the usefulness of a CDR lies not with its accuracy but with its ability to improve clinical outcomes and enhance the efficiency of care.[15] Even when a CDR demonstrates evidence of broad validation, this does not ensure that it will change clinical decision making, or that the changes it produces will result in better care. McGinn *et al.*[2] identified three explanations for the failure of a CDR at this stage. First, if clinician judgement is as accurate as a CDR informed decision, there is no benefit to its use. Second, the application of a CDR may involve cumbersome calculations or procedures which discourage clinicians from utilizing the CDR. Third, using the CDR may not be feasible in all environments or circumstances. In addition, we would include the reality that experimental studies may involve patients that are not entirely representative of those seen in routine care and that this may limit the actual value of a CDR. Therefore, to fully understand the utility of a CDR and its ability to improve healthcare delivery, it is necessary to undertake a pragmatic examination of its feasibility and impact when applied in an environment reflecting real world practice. This can be undertaken with different study designs such as randomized trials, cluster randomized trials, or other approaches such as examining the impact of a CDR before and after its implementation.

There has not been an impact analysis of the spinal manipulation CDR. Fritz and colleagues[16] did examine the impact of treatment based on the manipulation CDR by comparing the clinical outcomes (pain and disability) and healthcare utilization (number of treatment sessions, length of stay and healthcare costs) of CDR positive patients undergoing thrust manipulation, nonthrust manipulation or no manipulation. Patients receiving thrust or nonthrust manipulation experienced better clinical outcomes when compared to those receiving no manipulation. While the clinical outcomes did not differ between manipulation groups, patients receiving thrust manipulation utilized less healthcare resources than those receiving nonthrust manipulation. However, the study used a weaker, retrospective, case–control design. Additional impact studies using stronger research designs are needed.

## Appropriate interpretation of the diagnosis-based clinical decision rules

Two recent papers published in Chiropractic & Manual Therapies by Murphy and Hurwitz[17,18] highlight the sometimes confusing intersection between decision rules, patient classification and prediction of treatment outcome. The authors have previously published their work describing “diagnosis-based clinical decision rules” for patients with spinal pain.[19,20] As mentioned, the classification of patients with spinal pain is an important and potentially promising area of research activity and this diagnosis-based strategy is a welcome addition to other classification approaches.[21-25]

However, the author’s use of the phrase “diagnosis-based clinical decision rule” is noteworthy as it represents a departure from convention. A review of the development of the “diagnosis-based clinical decision rule” reveals no formal derivation, validation or analysis of impact. Instead, the criteria for this rule were derived from non-systematic literature reviews.[19,20] Therefore, the use of the phrase “diagnosis-based clinical decision rule” may be the cause of confusion as it implies the formal derivation and validation of a rule. Consequently, there is concern over the potential for a mistaken belief that the diagnosis-based decision rule represents a high level of evidence, while in its current form, it is an evidence-informed hypothesis. Therefore, it is not appropriate to refer to the criteria defining the diagnosis based classification approach as a clinical decision rule until the appropriately designed studies are undertaken.

In their recent publications, the authors have changed the terminology used to describe the diagnosis-based classification approach from “clinical decision rule” to “clinical decision guide.”[17,18] While the former implies a strict methodology of derivation, validation, and impact analysis, the later is an informal term perhaps more appropriate for the current state of the diagnosis-based classification approach and yet it is possible that the significance of this terminology change may be missed by the casual observer.

Moreover, distinctions between “decision rule,” “prediction rule,” “decision guide,” and “prediction guide”[26] can be subtle, and misunderstandings have potential to adversely impact clinical decision making. Although some may find such differences in terminology to be overly pedantic, these issues get to the heart of evidence appraisal and translation of evidence into practice and incorrectly identifying research findings as representing high level evidence (*e.g.*, a fully developed CDR) has potential to mislead clinicians and adversely affect patient care.

This perspective leads to two fundamental questions with respect to the diagnosis based classification approach:

1. What level of evidence does the diagnosis-based approach represent?

2. How should this evidence be used to inform patient care?

In its current state, the diagnosis-based clinical decision guide is a theoretical approach for classifying patients with neck or back pain. The authors have identified the potential classification criteria based upon narrative literature reviews.[19,20] While some of the individual studies identified in these reviews represent a high level of evidence, other studies underpinning the guide represent low level evidence. Furthermore, some aspects of the classification approach are hypothetical. The next logical step in the development of the diagnosis-based classification approach could be to undertake formal CDR derivation studies examining the multivariate relationships between potential predictor variables and treatment outcomes. Subsequently, the potential CDRs should be validated through the rigors of randomized trials. If broad validation of the CDRs were supported by experimental results, analyses of impact could then be undertaken. While it may be appropriate for CDR derivation study results to influence clinical decision making (*e.g.*, when very little evidence is available in a particular area or on a localized level where the impact on patient care can be monitored), widespread implementation of a CDR requires validation. Consequently, given the hypothetical nature of the diagnosis-based clinical decision rule (now termed clinical decision guide), its widespread implementation by clinicians is premature.

Although in its current state the diagnosis-based clinical decision guide represents a preliminary and untested approach, new and innovative developments in neck and back pain classification such as this should be welcomed and their development encouraged. However, it is also important to understand the appropriate context for interpreting this classification approach and recognize its limitations. Despite its potential to inform future efforts of subgrouping patients with neck or back pain who may preferentially respond to one or more forms of therapy, the diagnosis-based classification approach is not ready for clinical implementation. Nevertheless, we are hopeful that future advancements in and additional knowledge of patient classification hold promise for improving the quality of healthcare provided to patients with spinal pain and the diagnosis-based clinical decision approach is no exception.

## Competing interests

The authors declare that they have no competing interests.

## Authors’ contributions

JH and JF made substantial contributions regarding the conception and drafting of this manuscript. All authors read and approved the final manuscript.
